# Factors influencing COVID-19 vaccination intentions and the moderating effect of perceived behavioral control among pregnant women: a cross-sectional study applying the revised Theory of Planned Behavior

**DOI:** 10.4069/whn.2025.02.06

**Published:** 2025-03-28

**Authors:** So Youn Kim, Hee Sun Kang, Mijong Kim

**Affiliations:** 1 Red Cross College of Nursing, Chung-Ang University, Seoul, Korea; 2 Department of Nursing, Hannam University, Daejeon, Korea

**Keywords:** COVID-19, Intention, Pregnant Women, Theory of Planned Behavior, Vaccination

## Abstract

**Purpose:**

This study explored factors that influence coronavirus disease 2019 (COVID-19) vaccination intentions during pregnancy and examined the moderating effect of perceived behavioral control based on the revised Theory of Planned Behavior.

**Methods:**

This cross-sectional online survey recruited 227 Korean pregnant women from an online community. Data were collected from December 2021 to January 2022 and analyzed using independent t-test, analysis of variance, Pearson correlation coefficients, and multiple regression analysis. The PROCESS macro (model 1) and simple slope analysis were used to investigate the moderating effect of perceived behavior control.

**Results:**

Attitudes (β=.44, *p*<.001) and subjective norms (β=.36, *p*<.001) were identified as factors influencing COVID-19 vaccination intentions during pregnancy. In the final regression model, the total explanatory power of the variables was 44% (F=90.47, *p*<.001). The moderating effect of perceived behavioral control in the relationship between attitudes and vaccination intention was not statistically significant (B=0.07, *p*=.382). However, it showed a significant moderating effect in the relationship between subjective norms and vaccination intentions (B=0.06, *p*=.046). For simple slope analysis, perceived behavioral control was classified into three levels (low, moderate, and high), and the effect of subjective norms on vaccination intention was analyzed. Pregnant women with a high level of perceived behavioral control showed the strongest relationship (b=0.45, *p*<.001), indicating a moderating effect of perceived behavioral control.

**Conclusion:**

To increase vaccination intention among pregnant women, it is imperative to implement programs that focus on improving pregnant women’s attitudes, subjective norms, and perceived behavioral control toward vaccination, with particular attention to those with low perceived behavioral control.

## Introduction

The coronavirus disease 2019 (COVID-19) pandemic, which persisted for over 3 years since March 2020, has inflicted enormous global damage. By May 2023, when the World Health Organization (WHO) ended the public health emergency of international concern, approximately 765 million confirmed COVID-19 cases and around 6.9 million deaths had been reported worldwide [1,2]. Pregnant women—recognized as a high‐risk group due to their vulnerable immune systems—face an increased risk of severe disease progression if infected with COVID-19. They are more likely to require intensive care, mechanical ventilation, or experience fatal outcomes [3,4]. Additionally, COVID-19 infection during pregnancy has been associated with adverse outcomes such as preterm birth, cesarean delivery, and low birth weight, thereby jeopardizing fetal health [5,6].

In response to these urgent circumstances, COVID-19 vaccines were developed at an unprecedented pace. Pfizer and Moderna developed their vaccines in November 2020 and received emergency use authorization in December 2020, which facilitated rapid global distribution [7]. Although large-scale clinical trials involving tens of thousands of participants confirmed the vaccines’ efficacy and safety, vaccination rates among pregnant women remained generally low during this early phase. For instance, only approximately 16.3% of pregnant women in the United States were vaccinated [8], and global rates were around 27.5% [9]. This hesitancy primarily stemmed from the exclusion of pregnant women from the initial vaccine trials, leading to insufficient data on vaccine safety and efficacy during pregnancy.

As further evidence regarding vaccine effectiveness and safety in pregnant women emerged—and following extensive expert discussions—the WHO officially recommended COVID-19 vaccination for pregnant women in June 2021. Shortly thereafter, the U.S. Centers for Disease Control and Prevention (CDC) recommended vaccination for pregnant women, breastfeeding women, and those planning a pregnancy, based on data affirming vaccine safety for both mothers and fetuses [9,10]. In Korea, vaccination for pregnant women was recommended beginning in October 2021, with the Korea Disease Control and Prevention Agency disseminating promotional videos and informational materials that emphasized the vaccines’ safety and efficacy during pregnancy [10]. Despite these authoritative endorsements, many pregnant women remained hesitant; reported vaccination rates were approximately 31% in the United States [8], around 20% in the United Kingdom [11], and as low as about 10% in Korea by December 2021 [12]. Although ongoing data on vaccination trends are limited, these figures clearly demonstrate that many pregnant women have opted against vaccination despite official recommendations.

Several studies have demonstrated that COVID-19 vaccination is both safe and effective for pregnant women. For example, research in the United States found that infants born to vaccinated mothers experienced approximately a 60% reduction in the risk of COVID-19–related hospitalization [13]. Similarly, in the United Kingdom, the majority of maternal COVID-19 deaths among at least 40 reported cases occurred in unvaccinated women [14]. Key barriers to vaccination among pregnant women include anxiety arising from a lack of confidence in vaccine safety [15], concerns about potential adverse effects on both mother and fetus [16], and doubts regarding vaccine efficacy [17]. Additional challenges include limited access to vaccines and perceptions regarding the severity of COVID-19 [10]. Conversely, positive support from family and society [10] and convenient access to vaccination services [18] have been shown to increase vaccination intent.

Although numerous studies have explored pregnant women’s intentions to receive the COVID-19 vaccine—primarily via cross-sectional surveys [19,20] and meta-analyses [17,21]—these methods provide only a limited understanding of the underlying behavioral dynamics. Therefore, a more comprehensive investigation that incorporates individual behaviors, attitudes, and motivations is necessary.

The Theory of Planned Behavior (TPB) is a prominent model used to predict and explain individual actions [22] and has been widely applied across various fields [22,23]. According to TPB, the three key factors—attitudes, subjective norms, and perceived behavioral control—influence behavioral intentions, which in turn serve as the immediate precursors to actual behavior [24]. Due to practical constraints in measuring actual behavior, many TPB-based studies have used behavioral intentions as a proxy [25,26].

Moreover, Ajzen, the originator of TPB, later revised the theory to include the moderating effect of perceived behavioral control, suggesting that it interacts with attitudes and subjective norms to influence intentions [27,28]. Despite the extensive application of TPB in health-related research, studies that incorporate the moderating effect of perceived behavioral control within this revised framework remain scarce. In Korea, only a few studies have employed the revised TPB, such as in assessing parental intentions for human papillomavirus (HPV) vaccination among elementary school-aged sons [26] or predicting nurses’ intentions to care for COVID-19 patients [25].

Since 2000, major infectious diseases such as severe acute respiratory syndrome, Middle East respiratory syndrome, and the Zika virus have emerged, prompting researchers to warn that unpredictable outbreaks may become more frequent [29,30]. The recurring nature of these crises underscores the importance of developing safe and effective vaccines as well as receiving recommended vaccinations. It is crucial to ensure that high-risk groups, such as pregnant women, can make rapid and informed decisions regarding their health and disease prevention, which may also expand the role of nurses in women’s health care.

This study was conducted during a period when both the WHO and the U.S. CDC had officially recommended COVID-19 vaccination for pregnant women, following the accumulation of safety data and vaccine approval. In Korea, this period coincided with the launch of free vaccination programs. However, persistently low vaccination rates among pregnant women, despite endorsements from credible health authorities, have significant public health implications. To better prepare for future infectious disease outbreaks, it is essential to understand the behavioral responses observed during the recent pandemic and the impact of newly developed vaccines.

Therefore, this study aimed to identify the factors influencing pregnant women’s intentions to receive the COVID-19 vaccine and to examine the moderating effect of perceived behavioral control, based on the revised TPB. By evaluating vaccination intentions within this theoretical framework, the study is expected to both enhance our understanding and prediction of human behavior across diverse populations and contribute to the further development of health-related and nursing theories.

The purpose of this study was to determine the factors that influence pregnant women’s intentions to receive the COVID-19 vaccine and to investigate the moderating effect of perceived behavioral control. The study’s objectives were as follows:

1) To measure the levels of COVID-19 vaccination intention, attitudes, and subjective norms among the participants

2) To analyze differences in COVID-19 vaccination intention based on participants’ general and obstetric characteristics

3) To identify the factors influencing pregnant women’s intention to receive the COVID-19 vaccine

4) To examine the moderating effect of perceived behavioral control in the relationships between (a) vaccination attitudes and vaccination intention, and (b) subjective norms and vaccination intention

## Methods

**Ethics statement:** This study was approved by the Institutional Review Board of Chung-Ang University (No. 1041078-202110-HR-318-01). Informed consent was obtained from participants.

### Study design

**Figure 1.**
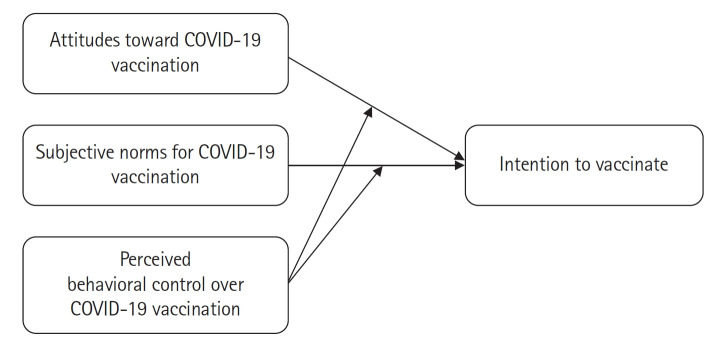
Framework of the present study based on the revised Theory of Planned Behavior (Ajzen, 2019). COVID-19: Coronavirus disease 2019.

This descriptive correlational study employed a cross-sectional survey to investigate the factors influencing COVID-19 vaccination intention among pregnant women. The study adhered to the STROBE guidelines (https://www.strobe-statement.org/) in reporting. The conceptual framework of the study, based on the revised TPB [23], is presented in Figure 1.

### Participants

The participants were pregnant women who met the inclusion criteria during the COVID-19 pandemic and provided voluntary consent. Inclusion criteria were: being at least 19 years old, currently pregnant, able to communicate in Korean, and having online access via devices such as a computer, tablet, or smartphone. Participants were recruited via the Korean online community “M** Baby” on the Naver Cafe platform. The sample size for regression analysis was determined using the G*Power 3.1.9.7 program, assuming an effect size of 0.08, a significance level of 0.05, and a power of 0.95, which yielded a minimum required sample size of 219. To account for a 15% dropout rate, 258 responses were collected. After excluding 30 participants who had already received the COVID-19 vaccine during pregnancy and one participant with incomplete responses, 227 responses were included in the final analysis.

### Measurements

#### COVID-19 vaccination intention

The intention to receive the COVID-19 vaccine was assessed using a single item. Participants rated their agreement on a 5-point Likert scale (1=“strongly disagree” to 5=“strongly agree”), with higher scores indicating a stronger intention to get vaccinated (possible range, 1–5).

#### COVID-19 vaccination attitudes

Kang et al.’s tool [31] (modified with the author’s approval) was used to evaluate pregnant women’s positive or negative attitudes toward COVID-19 vaccination. The instrument consists of 14 items rated on a 5-point Likert scale (1=“not at all” to 5=“strongly agree”) across three domains: safety, efficacy, and necessity. The total score ranges from 14 to 70, with higher scores reflecting a more positive attitude toward receiving the COVID-19 vaccine during pregnancy. In Kang et al.’s original study [31], the tool demonstrated a Cronbach’s α of .85; in the current study, it was .84.

#### Subjective norms regarding COVID-19 vaccination

To measure subjective norms regarding COVID-19 vaccination, we adapted a tool originally developed by Lee [26] for measuring subjective norms related to HPV vaccination, with the author’s permission. The scale comprises two items rated on a 7-point Likert scale (1=“strongly disagree” to 7=“strongly agree”) and higher scores (possible range, 2–14) indicate greater perceived pressure from important others regarding COVID-19 vaccination. Cronbach’s α was .74 in Lee’s study [26] and .88 in the current study.

#### Perceived behavioral control regarding COVID-19 vaccination

Perceived behavioral control was measured using a tool originally developed by Lee [26] for HPV vaccination, modified with the author’s permission. This instrument assessed the extent to which pregnant women perceived receiving the COVID-19 vaccine as easy or difficult. The scale includes 4 items rated on a 7-point Likert scale (1=“strongly disagree” to 7=“strongly agree”). Higher scores (possible range, 4–28) indicate greater confidence and an easier perception of vaccination. Cronbach’s α was .89 in Lee’s study [26] and .86 in the current study.

### Data collection

Data were collected from December 15, 2021, to January 15, 2022. Recruitment involved explaining the study’s purpose, duration, location, target population, and content to the managers of a Korean online community for pregnant women and mothers, “M** Baby” cafe, and obtaining permission to collect data from its members. A recruitment notice was posted on the forum, and a survey link was shared with members who voluntarily expressed interest in participating. The online survey provided detailed information about the study’s purpose and methods, assured participants that the data would be used solely for research purposes, and informed them of their right to withdraw at any time. The survey commenced only after participants provided their consent. As a token of appreciation, all participants received a small incentive worth 5,000 Korean won (KRW; approximately 5 US dollars).

### Data analysis

The collected data were analyzed using SPSS for Windows version 26.0 (IBM Corp., Armonk, NY, USA) and the PROCESS macro. Frequencies and percentages were computed to describe participants’ general, obstetric, and COVID-19-related characteristics. Means and standard deviations (SDs) were calculated for COVID-19 vaccination intention, vaccination attitudes, subjective norms, and perceived behavioral control. To examine differences in vaccination intention based on participants’ characteristics, the independent t-test and analysis of variance were performed. Pearson correlation coefficients were used to assess relationships among COVID-19 vaccination intention, vaccination attitudes, subjective norms, and perceived behavioral control. Finally, multiple linear regression analysis was conducted to evaluate the effects of vaccination attitudes, subjective norms, and perceived behavioral control on vaccination intention.

Furthermore, the moderating effect of perceived behavioral control on the relationships between vaccination intention, vaccination attitudes, and subjective norms was examined using the PROCESS macro (Model 1) proposed by Hayes [32]. When a significant moderating effect was identified, a simple slope analysis was conducted. In this analysis, participants were categorized into three groups based on their perceived behavioral control scores: low (mean–1 SD), moderate (mean), and high (mean+1 SD). The slopes between the independent and dependent variables were then compared across these groups.

## Results

### General and obstetric characteristics of participants

Table 1 summarizes the general and obstetric characteristics of the 227 study participants. Among these participants, 159 (70.0%) were under 35 years old, with an average age of 33.0±3.0 years. The majority held a college degree or higher, with 192 participants (84.6%) being college graduates and 23 (10.1%) holding an advanced degree. Furthermore, 140 participants (61.7%) reported earning a monthly income of 5 million KRW (approximately 5,000 US dollars) or more, followed by 61 participants (26.9%) earning between 3 and 5 million KRW (3,000–5,000 US dollars). As the average monthly household income in Korea in 2021 was approximately 5.1 million KRW [33], the most common income bracket among participants aligned with the national average. Regarding employment, the majority of participants (n=137, 60.4%) were not employed. Additionally, 182 participants (80.2%) were first-time mothers, and the second trimester was the most common stage of pregnancy, with 128 participants (56.4%) (Table 1).

### COVID-19 vaccination intention according to general and obstetric characteristics

No significant differences were found in COVID-19 vaccination intention according to participants’ general or obstetric characteristics (Table 1).

### Descriptive statistics of study variables

The average score for intention to receive the COVID-19 vaccine during pregnancy was 2.67±1.21 on a 5-point scale. The score for vaccination attitudes was neutral point with an average of 38.37±9.48, and the subjective norms score was neutral point with an average of 7.91±2.10. The perceived behavioral control score averaged 19.93±5.30, greater than neutral point (Supplement Table 1).

### Relationships among intention to vaccinate, attitudes, subjective norms, and perceived behavioral control

Pregnant women’s attitudes toward COVID-19 vaccination were moderately and significantly positively correlated with vaccination intention (r=.57, *p*<.001). Likewise, subjective norms were moderately and significantly positively correlated with vaccination intention (r=.52, *p*<.001) (Table 2).

### Factors influencing COVID-19 vaccination intention

Table 3 presents the regression analysis results for factors influencing COVID-19 vaccination intention among pregnant women. Multicollinearity was assessed, with all tolerance values at 0.88 (above the 0.1 threshold) and all variance inflation factors at 1.13 (below the threshold of 10), indicating no multicollinearity issues. The Durbin-Watson test produced a value of 1.91, close to 2, confirming that the residuals were independent and that the assumptions of normality and homoscedasticity were satisfied. The regression model was statistically significant (F=90.47, *p*<.001) and explained 44% of the variance in vaccination intention. Among the factors examined, vaccination attitudes (β=.44, *p*<.001) and subjective norms (β=.36, *p*<.001) had significant positive effects on vaccination intention, with attitudes showing the strongest influence (Table 3).

### Moderating effect of perceived behavioral control

Table 4 presents the results of the analysis examining the moderating effect of perceived behavioral control on the relationships between (a) vaccination attitudes and vaccination intention and (b) subjective norms and vaccination intention among pregnant women.

The regression model assessing the moderating effect of perceived behavioral control on the relationship between vaccination attitudes and vaccination intention was statistically significant (F=41.87, *p*<.001). However, the interaction term between attitudes and perceived behavioral control was not significant (B=0.07, *p*=.382). In contrast, the regression model testing the moderating effect of perceived behavioral control on the relationship between subjective norms and vaccination intention was also statistically significant (F=29.57, *p*<.001), with a significant interaction term (B=0.06, *p*=.046)

**Figure 2.**
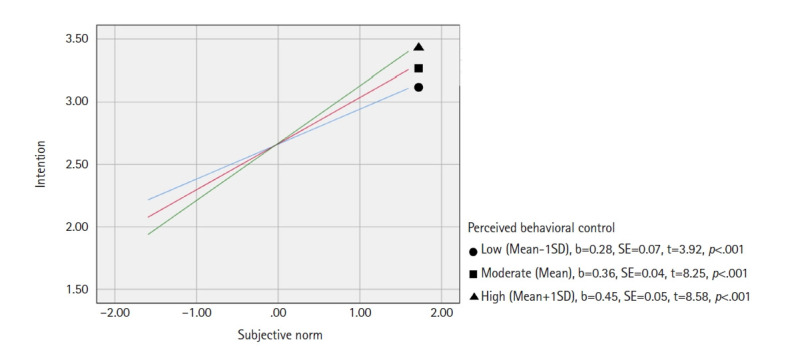
Simple slope analysis: moderation effect of perceived behavioral control on the relationship between subjective norm and vaccination intention.

A simple slope analysis was conducted to further examine how subjective norms influenced vaccination intention at different levels of perceived behavioral control (Figure 2). Participants were divided into three groups based on their perceived behavioral control scores: low (mean–1 SD), moderate (mean), and high (mean+1 SD). The analysis revealed that the high perceived behavioral control group had the steepest slope (effect=0.45, *p*<.001), followed by the moderate group (b=0.36, *p*<.001), while the low perceived behavioral control group showed the shallowest slope (b=0.28, *p*<.001) (Figure 2).

## Discussion

This study aimed to identify the factors influencing pregnant women’s intentions to receive the COVID-19 vaccine based on the revised TPB. The findings indicate that vaccination attitudes and subjective norms are significant predictors of vaccination intention. Consistent with the revised TPB, perceived behavioral control moderates the relationship between subjective norms and vaccination intention.

In this study, the average COVID-19 vaccination intention among pregnant women was at a moderate level (2.67±1.21), which contrasts with previous research—for example, a TPB-based study reporting an average score of 3.08±0.91 for pregnant women’s intention to receive the influenza vaccine on the same scale [31]—suggests that the vaccination intention in this study is notably lower. A meta-analysis of 12 studies on COVID-19 vaccination intention among pregnant women revealed rates ranging from 19% in Africa to 47% in Oceania, underscoring the influence of regional pandemic conditions and national policies [34]. Overall, while COVID-19 vaccination intentions among pregnant women were generally low, those with a history of receiving influenza or Tdap (tetanus, diphtheria, and pertussis) vaccine demonstrated significantly higher vaccination intentions. Another meta-analysis using TPB to systematically review factors affecting COVID-19 vaccination intention reported an average intention rate of 73.1%, with a wide range from 31% to 88.8% depending on the region and timing of the study during the pandemic [35]. These findings suggest that the vaccination intention level observed in this study is somewhat lower than global averages, potentially reflecting the safety-conscious attitudes prevalent among pregnant women in Korea—a phenomenon warranting further investigation.

The results confirmed that vaccination attitudes are a significant predictor of vaccination intention, meaning that the more positively pregnant women view the COVID-19 vaccine, the greater their intention to get vaccinated. Similarly, previous studies on influenza vaccination among pregnant women [31] and on nurses’ intentions to care for COVID-19 patients have identified attitudes as a key determinant. A meta-analysis of TPB-based studies on COVID-19 vaccination intention [34] further highlighted attitudes as the most potent predictor among the examined variables. These findings reaffirm that within the TPB framework, attitudes are a robust predictor of vaccination intention. Despite evidence from multiple studies showing that COVID-19 vaccination during pregnancy reduces the risk of infection, shortens hospital stays, and does not significantly impact maternal-fetal complications [10,36], negative attitudes toward the vaccine remain prevalent among pregnant women. Therefore, providing reliable information and positive messaging is essential for fostering more favorable attitudes. Pregnant women’s perceptions of the COVID-19 vaccine are influenced by their beliefs about its efficacy and safety, as well as by their overall health concerns. Women with positive attitudes are more likely to recognize the benefits and effectiveness of vaccination, thereby increasing their intention to get vaccinated. This aligns with previous research [31,35] and underscores the importance of accurate, trustworthy information and educational initiatives aimed at promoting a positive outlook on vaccination. Given that pregnant women are particularly concerned about fetal health and potential risks associated with the vaccine, healthcare providers and public health agencies must offer scientifically grounded information to address these concerns. Emphasizing the vaccine’s ability to protect both maternal and fetal health can help foster a more positive attitude. Given that misinformation and fears of adverse effects often deter vaccination, it is necessary to communicate new vaccine developments promptly and effectively through mass media, supported by expert guidance tailored to pregnant women’s needs.

Subjective norms were also identified as an important predictor of vaccination intention, with their effect significantly moderated by perceived behavioral control. Subjective norms reflect an individual’s tendency to conform to the expectations of influential others [23]. For pregnant women, these norms are shaped by family, friends, healthcare providers, and broader societal expectations. The finding that subjective norms strongly predict vaccination intention suggests that when close contacts—such as spouses, family members, and friends—express positive attitudes toward the COVID-19 vaccine, pregnant women are more likely to intend to get vaccinated. This result underscores the need to strengthen the social support systems surrounding pregnant women to encourage vaccination. Additionally, recommendations from healthcare providers can significantly influence subjective norms. Previous research has shown that when healthcare professionals recommend vaccination and explain its benefits and safety, pregnant women are more likely to trust the process and develop higher vaccination intentions [10,37]. This implies that during pregnancy, women tend to prioritize the opinions of healthcare experts when making vaccination decisions. Therefore, healthcare providers should routinely assess vaccination status during prenatal visits and offer comprehensive, evidence-based information to support informed decision-making. Moreover, support and encouragement from family and friends are essential in influencing vaccination decisions. If a pregnant woman’s spouse or family expresses concerns about the safety of rapidly developed vaccines, her vaccination intention may decrease [16,20]. To address this, including family members—particularly spouses—in educational programs could be beneficial. Providing accurate information through social media or establishing a hotline for inquiries may also help alleviate vaccination concerns.

Furthermore, pregnant women with high perceived behavioral control showed a more pronounced influence of subjective norms on their vaccination intention. This suggests that those with greater perceived behavior control are more responsive to social expectations and pressures, thereby reinforcing their vaccination intentions. In contrast, pregnant women with low perceived behavioral control are less influenced by subjective norms, indicating that interventions should be tailored to address these differences. In practice, when providing vaccination-related education, if a pregnant woman is identified as having low perceived behavioral control, it may be more effective to emphasize content aimed at improving attitudes rather than relying solely on subjective norms.

Perceived behavioral control reflects a pregnant woman’s confidence in her ability to successfully complete the vaccination process. Although perceived behavioral control did not have a statistically significant direct effect on vaccination intention in this study, it indirectly influences vaccination intention by moderating the impact of subjective norms. Consequently, perceived behavioral control alone may not be a strong predictor of vaccination intention, which supports the revised TPB. Previous studies have found that perceived behavioral control is a strong predictor of both behavioral intention and actual behavior [35]; however, our findings indicate that, among pregnant women, it does not directly predict COVID-19 vaccination intention. This result is consistent with a study conducted among couples preparing for pregnancy in China, where perceived behavioral control did not serve as a key determinant linking COVID-19 vaccination intention to actual vaccination behavior [16]. These results offer a new perspective by showing that perceived behavioral control moderates the relationship between subjective norms and vaccination intention, thus supporting the revised TPB.

A detailed analysis of the moderating effect of perceived behavioral control—specifically, its influence on the relationship between subjective norms and vaccination intention—revealed a consistently positive relationship across groups with high, moderate, and low perceived behavioral control, such that higher subjective norms correspond to greater vaccination intentions. Notably, the group with high perceived behavioral control exhibited the steepest slope for the relationship between subjective norms and vaccination intention, whereas the group with low perceived control showed the shallowest slope. In other words, when pregnant women have low perceived behavioral control, vaccination intention does not vary significantly with different levels of subjective norms; however, when perceived behavioral control is high, the impact of subjective norms on vaccination intention becomes markedly stronger. These findings suggest that social pressure has less influence on vaccination intention among those who view the vaccination process as difficult and a greater impact among those who view it as easy. Therefore, when implementing intervention programs to promote vaccination among pregnant women, healthcare providers should first assess each woman’s level of perceived behavioral control. For those with low perceived control, it may be more effective to emphasize content designed to improve attitudes rather than relying solely on subjective norms. For example, involving a spouse or family members in the program could increase vaccination intention among women with low perceived control. If family participation is not feasible, the role of healthcare professionals and the creation of a positive atmosphere among peer groups becomes particularly important, as these women are highly influenced by subjective norms and may decide to get vaccinated more readily when exposed to positive peer pressure.

The positive effect of subjective norms on vaccination intention was more pronounced when perceived behavioral control was high. Although perceived behavioral control did not moderate the relationship between vaccination attitudes and vaccination intention, it played a crucial moderating role in the relationship between subjective norms and vaccination intention. Therefore, boosting perceived behavioral control among pregnant women may be essential for increasing COVID-19 vaccination intention. These findings suggest that pregnant women with low perceived behavioral control require additional support and education when making important health decisions such as vaccination. The fact that the influence of subjective norms on vaccination intention was stronger as perceived behavioral control increased indicates that interventions focused on enhancing perceived behavioral control could improve vaccination intention among pregnant women. Interventions should aim to enhance attitudes, reinforce subjective norms, and increase perceived behavioral control, with particular emphasis on those with lower perceived behavioral control.

The limitations of this study are as follows: First, the participants were recruited through convenience sampling from an online community, which limits the generalizability of the results. Second, the study measured vaccination intention as the final dependent variable rather than actual vaccination behavior, which may not always correlate perfectly. Nonetheless, numerous previous studies have shown that intention is a direct determinant of behavior. Future research should conduct longitudinal studies to determine whether intention ultimately translates into actual vaccination. Despite these limitations, this study provides valuable foundational data for understanding pregnant women’s COVID-19 vaccination intentions in the context of a global pandemic.

In conclusion, our revised TPB-based analysis of the factors influencing COVID-19 vaccination intentions and the moderating effect of perceived behavioral control among pregnant women indicates that promoting positive vaccination attitudes and reinforcing subjective norms and social support systems are essential for enhancing vaccination intention. Moreover, the positive effect of subjective norms is significantly stronger when perceived behavioral control is high. Therefore, to boost vaccination intention among pregnant women, interventions should focus on improving attitudes, reinforcing subjective norms, and increasing perceived behavioral control—particularly among those with low perceived behavioral control.

## Figures and Tables

**Table 1.** COVID-19 vaccination intention according to general and obstetrical characteristics (N=227)

Variable	Categories	Mean±SD or n (%)	Intention to vaccinate	
			Mean±SD	t or F (*p*)
Age (year)		33.0±3.0		
	<35	159 (70.0)	2.74±1.17	1.35 (.179)
	≥35	68 (30.0)	2.50±1.28	
Education	High school	12 (5.3)	2.08±1.38	2.07 (.128)
	College	192 (84.6)	2.67±1.17	
	>College	23 (10.1)	2.96±1.40	
Monthly income (KRW)	<3 million	26 (11.4)	2.38±1.17	1.06 (.348)
	3–5 million	61 (26.9)	2.61±1.32	
	>5 million	140 (61.7)	2.74±1.17	
Job	Yes	90 (39.6)	2.66±1.24	–0.09 (.923)
	No	137 (60.4)	2.67±1.20	
Gravidity	1st	182 (80.2)	2.70±1.21	0.47 (.624)
	2nd	38 (16.7)	2.53±1.25	
	≥3rd	7 (3.1)	2.43±0.98	
Pregnancy trimester	First	36 (15.9)	2.56±1.21	2.07 (.129)
	Second	128 (56.4)	2.80±1.13	
	Third	63 (27.7)	2.44±1.34	

COVID-19: Coronavirus disease 2019; KRW: Korea won (1 million KRW is approximately 1,000 US dollars).

**Table 2.** Relationships among intention to vaccinate, attitudes, subjective norms, and perceived behavioral control (N=227)

Variable	r (*p*)		
	Intention to vaccinate	Attitudes	Subjective norms
Intention to vaccinate	1		
Attitudes	.57 (<.000)	1	
Subjective norms	.52 (<.000)	.34 (<.000)	1
Perceived behavioral control	–.02 (.687)	.25 (<.000)	–.01 (.886)

**Table 3.** Factors influencing COVID-19 vaccination intention (N=227)

Variable	B	SE	β	t	*p*	TOF	VIF
(Constant)	0.40	0.25		–1.59	.112		
Attitudes	0.79	0.09	.44	8.43	<.001	.88	1.13
Subjective norms	0.27	0.04	.36	6.96	<.001	.88	1.13
F (*p*)	90.47 (<.001)						
Adjusted R^2^	.44						
Durbin-Watson	1.91						

COVID-19: Coronavirus disease 2019; TOF: tolerance of fit; VIF: variance inflation factor.

**Table 4.** Moderation effect of perceived behavioral control

Variable	B	SE	t	*p*	F (*p*)	R^2^
Attitudes	1.04	0.11	8.69	<.001		
Perceived behavioral control	–0.15	0.05	–2.87	.004		
Attitude×perceived behavioral control	0.07	0.08	0.87	.382	41.87 (<.001)	.36
Subjective norms	0.36	0.04	8.25	<.001		
Perceived behavioral control	0.00	0.05	0.05	.960		
Subjective norms×perceived behavioral control	0.06	0.03	2.00	.046	29.57 (<.001)	.28

## Supplementary materials

Further details on supplementary materials are presented online (available at https://doi.org/10.4069/whn.2025.02.06).

Supplementary Table 1. Descriptive statistics of study variables: intention to vaccinate, attitudes, subjective norms, and perceived behavioral control (N=227)
