# Supplementary material for: Factors influencing COVID-19 vaccination intentions and the moderating effect of perceived behavioral control among pregnant women: a cross-sectional study applying the revised Theory of Planned Behavior
Source: Womens Health Nurs. 2025 Mar 28;31(1):34–45. doi: 10.4069/whn.2025.02.06 (PMC12010802; doi:10.4069/whn.2025.02.06)
Supplement: Supplementary Table 1. — Descriptive statistics of study variables: intention to vaccinate, attitudes, subjective norms, and perceived behavioral control (N=227) [file whn-2025-02-06-Supplementary-Table-1.pdf]

Supplementary Table 1. Descriptive statistics of study variables: intention to vaccinate, attitudes, subjective norms, and perceived behavioral control (N=227)

Variable	Mean ± SD	Possible range	Data range
Intention to vaccinate	2.67 ± 1.21	1–5	1–5
Attitudes	38.37 ± 9.48	14–70	18–70
Subjective norms	7.91 ± 2.10	2–14	2–14
Perceived behavioral control	19.93 ± 5.30	4–28	4–28
